# Uncovering three-dimensional gradients in fibrillar orientation in an impact-resistant biological armour

**DOI:** 10.1038/srep26249

**Published:** 2016-05-23

**Authors:** Y. Zhang, O. Paris, N. J. Terrill, H. S. Gupta

**Affiliations:** 1 Queen Mary University of London, School of Engineering and Material Science, London, E1 4NS, UK; 2 Institute of Physics, Montanuniversitaet Leoben, Leoben, Austria; 3 Diamond Light Source, Harwell Science and Innovation Campus, Harwell, UK

## Abstract

The complex hierarchical structure in biological and synthetic fibrous nanocomposites entails considerable difficulties in the interpretation of the crystallographic texture from diffraction data. Here, we present a novel reconstruction method to obtain the 3D distribution of fibres in such systems. An analytical expression is derived for the diffraction intensity from fibres, explaining the azimuthal intensity distribution in terms of the angles of the three dimensional fibre orientation distributions. The telson of stomatopod (mantis shrimp) serves as an example of natural biological armour whose high impact resistance property is believed to arise from the hierarchical organization of alpha chitin nanofibrils into fibres and twisted plywood (Bouligand) structures at the sub-micron and micron scale. Synchrotron microfocus scanning X-ray diffraction data on stomatopod telson were used as a test case to map the 3D fibre orientation across the entire tissue section. The method is applicable to a range of biological and biomimetic structures with graded 3D fibre texture at the sub-micron and micron length scales.

A ubiquitous characteristic of biological structural materials is the presence of a three-dimensional organized network of crystalline or partly crystalline nanofibres (often inside a less structured matrix). These include chitin fibrils in arthropod cuticle^1^, the spiralling fibrillar structure in wood cells^2^ or bone lamellae^3^, and actin fibrils in the cytoskeleton^4^. These networks are usually far from spatially homogeneous, but vary at multiple length scales (e.g. at the nanometre and micrometre scale), and these multiscale gradients are often key to the function and performance of the entire unit^5^. In synthetic composites, networks of nanofibres have been extensively used for structural reinforcement due to their large surface area to volume ratio and controllable surface functionalities^6^,^7^,^8^, as well as in conductive materials^9^, tissue engineering^10^,^11^, high-strength energy storage materials^12^,^13^ and sensors^7^,^14^. It is believed that the functionalities of both the natural and synthetic materials are highly dependent on the orientation, degree of crystallization and amount of the nanofibres^15^. Composite elastic moduli increase up to a factor of five when the carbon nanofibres changes from random orientation and perfect alignment in a polymer matrix^16^. Micro-mechanical tests also indicate that the elastic modulus variation corresponds to orientation changes of the fibre plane within the lamella structure of biological materials^17^,^18^. These structural alterations have significant effects on properties, for example better conductivity of synthetic composites can be obtained from highly aligned nanofibres^10^, and the elastic moduli of bone lamellae are predominantly determined by collagen fibril orientation^18^,^19^. Reconstructing – in a non-destructive and quantitative manner – the three dimensional orientation, crystallographic structure and supramolecular morphology of such nanofibrous composites is thus a question of very wide technological and scientific relevance in both synthetic and biological materials. Such a task, however, is technically challenging due to the small length scales both of the constituent units (1–100 nm) and over which the variation is to be mapped (hundreds of nm to several ten μm).

Conventional X-ray powder diffraction techniques have long been used in determining crystallographic lattice structure of constituent elements of composites with fibrous components such as collagen, DNA, cellulose, chitin, carbon nanofibres and synthetic polymers^20^,^21^,^22^. Aspects of the structure beyond the parameters of the unit cell, however – such as fibre orientation and texture, and microscale gradients in these parameters – introduce increasing complexity in the interpretation of the two dimensional diffraction patterns, some of which cannot be addressed within current techniques. A class of advanced technical materials where this interpretation is especially important is in the category of high strength, lightweight impact-resistant composites^23^ - both biological and synthetic – where controlled variation of fibre orientation, gradients in fibre texture and crystallinity play an important role (amongst other factors) in achieving their excellent dynamic mechanical properties. The cuticle of arthropods – especially species adapted to extremely high loading rates such as stomatopods – is an excellent example of high dynamic impact resistance, believed to be achieved with a hierarchical structure design and composed of crystalline alpha chitin nanofibres, proteins and minerals^24^,^25^,^26^. It is therefore an ideal model system, where determining 3D nanofibre texture and orientation will not only enable the development of a novel reconstruction method but will also serve as inspiration for research in biomimetic composites.

Arthropod cuticle holds several lessons for materials scientists attempting to replicate the high mechanical competence of natural biological materials like cellulose^27^, mineralized collagen^28^ and chitin^29^,^30^. Through a combination of a stiff phase of alpha-chitin fibrils with a more extensible but tough protein matrix, a variable degree of water content, and a stiff mineral phase, the nanoscale structure achieves both stiffness and high toughness, a feature common to other natural composites^1^. As a biological composite, cuticle is both renewable and regenerated, with moulting cycles achieved over weeks^31^. Further, in many species, cuticle (particularly crustacean cuticle) achieves very high toughness and impact resistance^30^,^32^ via evolutionary adaptation to predation and intraspecific fighting (and in some intertidal species, to resist strong waves impacting rocky shores). Finally, mineralized cuticle is built in a layer-wise/scaffold manner to enable progressive deposition of layers of a chitin/protein/mineral matrix away from the interior soft tissue^31^, together with a network of pore canals running perpendicular to the surface which transport the components of the stiff reinforcing mineral phase^33^. The basic building block is combined (*via* cell-directed self-assembly) into elements at multiple hierarchical levels (Fig. 1). Generic types of these structural elements include twisted plywood stacks of mineralized fibrils (known as Bouligand structures^34^) at the microscale, which differ from similar structures in bone by the presence of a perforating network of pore-canals supplying nutrients (Fig. 1c3–c5)^30^,^33^,^35^. The combination of the pore canals with the in-plane Bouligand stacks leads to a structural arrangement in crustacean exoskeleton which has been likened to a “honeycomb” structure by researchers^32^,^33^. These multiple hierarchical levels are summarized in Fig. 1b1–b4^30^. Particular examples of high impact resistance occur in certain stomatopod (mantis shrimp) species (such as *Odontodactylus scyllarus*), which have developed a striking club (dactyl^26^,^36^) for predation and a ribbed shield (telson^37^) for defence, both of which are mineralized cuticular structures capable of resisting high rates of load without structural damage. These properties arise from specific structural mechanisms at small scales; for example, it has been shown that for the dactyl a combination of quasi-plastic contact mechanical response, associated with sliding and rotation of fluorapatite nanorods in the outer part of the organ^24^, together with the Bouligand architecture in the inner region of the dactyl, play important roles in the high impact resistance and mechanical quality^26^.

To reconstruct the 3D fibrillar morphology in such graded biological composites, combination of focussed synchrotron microfocus X-ray beams^20^ with model-based reconstruction of the X-ray diffraction and scattering signal from very small volumes is a promising approach. Real-space imaging methodologies at the 0.1–10 nm scale (e.g. scanning electron microscope (SEM), transmission electron microscope (TEM) and scanning probe microscopy (SPM)) are limited to localized measurements of fibre orientation at the material surface and provide little information on the third (depth) dimension. Conversely, inverse-space imaging of molecular and supramolecular structure are usually performed using X-ray texture analysis via pole figure measurements^38^,^39^,^40^, Schulz reflection method^41^, and parallel beam X-ray diffraction^42^. While these measurements provide full three-dimensional information of the distribution of crystallographic axes, this information is averaged over volumes of the order of hundreds of microns if not millimetres, due to the spatial rotation inherent in such methods. Such methods are therefore incapable of locally resolving 3D fibrillar structure in microvolumes and over the small scales (typically upto a few tens of microns) characteristic of structural gradients in biological materials. Many efforts have been made to develop analytical methods to relate the orientation of crystalline fibres orientations to the diffraction patterns collected on the detectors during experiments^43^. Principal fibre direction has been determined with high energy X-ray diffraction in bone, cellulose and cuticle^2^,^3^,^44^,^45^ but not structures replicating the full three-dimensional fibre architecture. A mathematical equation relating orientation of cellulose crystallites in a single flax fibre with the azimuthal diffraction intensity distribution has been previously proposed^46^. The model, however, assumed that the fibres were located in the plane parallel to the X-ray beam direction. This may not always be the case in two dimensional scanning of a previously unknown material in which the orientation of fibre planes varies in different locations. Lastly, 3D reconstruction via analytic models should be preferably be relatively inexpensive computationally (rather than “brute force” optimization methods), in order to obtain the reconstructed morphology in a near-real time manner, a consideration especially relevant in microfocus synchrotron scanning experiments where large sample areas (relative to beam size) are carried out routinely.

Here, we present such a reconstruction method to determine the full 3D lamellar architecture of chitin fibre orientations in the case of a natural biological armour – the telson of the stomatopod (mantis shrimp)^37^.

## Development of Analytical Model

### 3D model for 2D diffraction pattern of chitin fibrils

**Figure 1:**
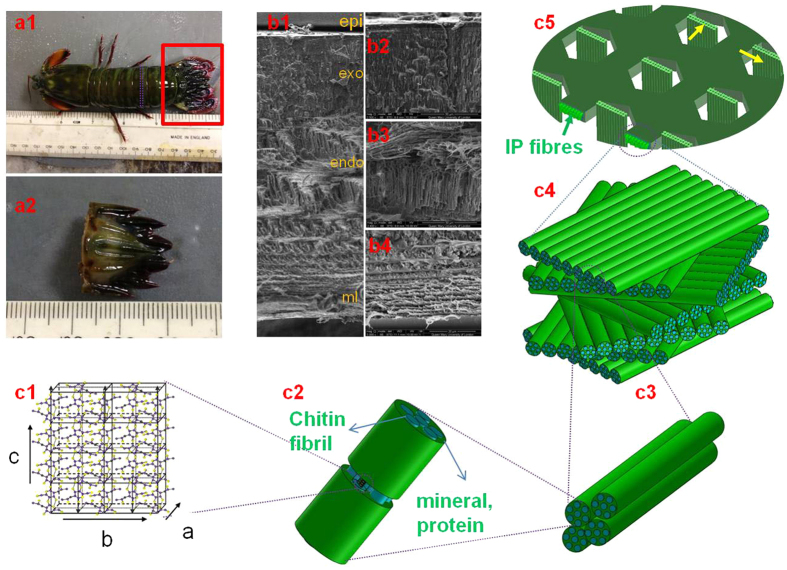
Hierarchical structure of cuticle of stomatopod (mantis shrimp). (**a**) Morphology of the stomatopod (**a1**). The red rectangle shows the telson sample (**a2**) used as sample for synchrotron scanning-WAXD test after dissection; (**b**) SEM image (**b1**) from a cross section of the cuticle shows the lamellar structure. The epicuticle (epi), exocuticle (exo), endocuticle (endo) and membranous (ml) layers are indicated. The three higher magnification SEM images show different fibre stack intensities across exocuticle (**b2**), endocuticle (**b3**) and membranous layer (**b4**); (**c**) The hierarchical structure of cuticle starts from N-acetyl-glucosamine molecules arranged in an orthorhombic crystal structure (**c1**) (image taken from^50^) with 18 to 25 chitin molecules wrapped with proteins form nanofibrils (blue cylinder in **c2**), together with minerals, the nanofibrils cluster into nanofibres (**c3**), the chitin fibre-protein planes stack into plywood structure with fibre-protein planes rotated around the normal axis of the cuticle (**c4**). The plywood (Bouligand) structures with in-plane (IP) fibres together with the out-plane fibres (OP) running through the pore-canal system in the cuticle (**c5**).

**Figure 2:**
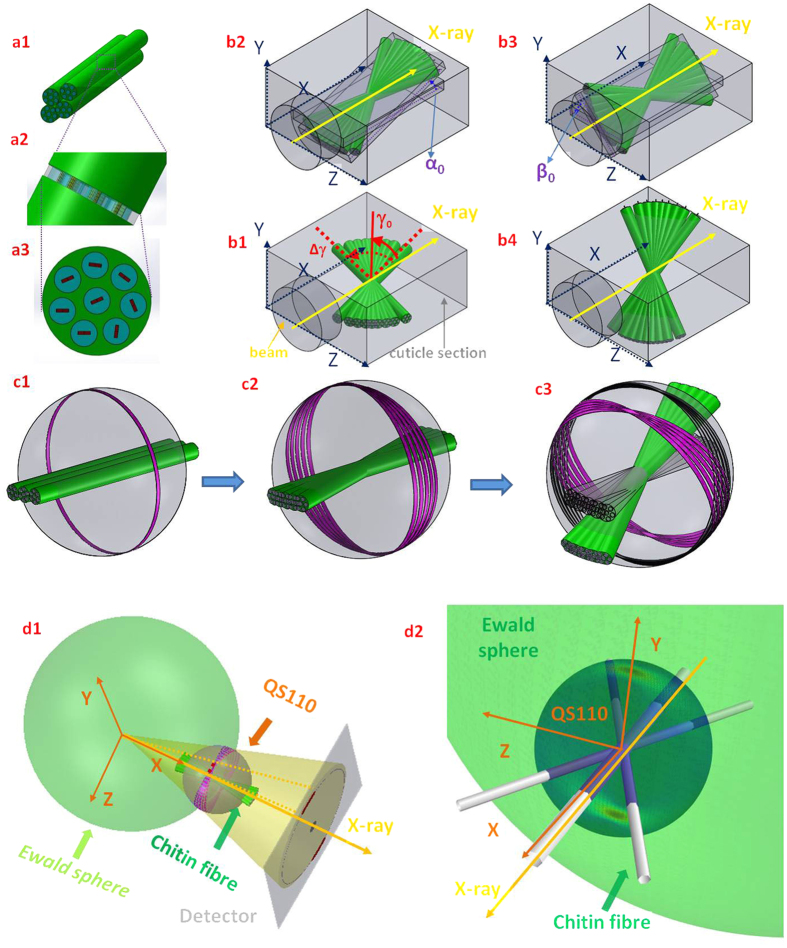
Relation between chitin fibre arrays, fibre symmetry and the diffraction condition. (**a**) The randomly oriented reciprocal 110 vectors from each single fibre around the mineralized chitin fibre axis result in smearing of reflections into reflection rings in the sphere described by rotating the reciprocal (110) over all possible 3D directions (*hereafter described as QS110 with QS shorthand for q-vector sphere*) of the fibre (**a1–a3**), i.e. a fibre symmetry condition (**c1**). (**b**) Schematics illustrating the fibre orientation distribution parameters (*γ*_*0*_, Δ*γ*_*0*_) within a single lamella plane parallel to the beam (**b1**). The variation of fibre orientations will generate multiple intersected rings on *QS110*, and the rings from continuously distributed fibre plane then smear into a reflection band on the sphere (**c2**). The yellow arrow in **b1** denotes the direction of the incident beam. The fibre planes may tilt in 3D (*α,* **b2**), (*β*, **b3**) or a combination of both angles (**b4**) which results in the tilting of the reflection band (**c3**) on *QS110*. **d1):** Sketch of the fibre diffraction geometry, with the Ewald’s sphere shown on left and *QS110* on right. The intensity on the intersection ring of the Ewald’s sphere and *QS110* will contribute to the diffraction signal. (**d2**): The intersection of the Ewald’s sphere and *QS110* plotted for a specific 3D fibre orientation (the fibre orientation is schematically shown as three white fibres plotted along the main fibre direction and at ± the half width at half maximum away from the main fibre direction). The incident beam directions are indicated in both (**d1**) and (**d2**) using yellow arrows. Here, the regions of high scattered intensity appear as distinct red streaks toward the upper and lower parts of *QS110*.

The c-axis of the orthorhombic unit cell of chitin has been found to be parallel to the longitudinal axis of the chitin fibril^47^. Hence, the orientation of the c-axis can be used as a proxy for the chitin fibril orientation (Fig. 1c1). Fibre symmetry of the unit cell of chitin around the c-axis in the fibril is assumed (Fig. 2a1–a3). In such a condition, the 3D reciprocal lattice of the chitin unit cell is rotated around the c-axis, leading to rings of equal intensity in reciprocal space rather than discrete spots, analogous to the case for cellulose microfibrils^48^,^49^. The measured wide-angle X-ray diffraction (WAXD) pattern is the projection on a 2D detector of the intersection of the Ewald’s sphere with the collection of rings, as shown in Fig. 2d1. Standard texture measurements (rotating the sample about Euler axes)^26^,^40^ do not suffice to obtain the spatially resolved 3D intensity distribution for micro-structurally heterogeneous materials like the telson, because the volume hit by the X-ray beam is not constant for different rotation angles.

Consider initially a fibre oriented approximately along the q_x_-axis (i.e. a fibre oriented parallel to the beam direction). We use the equatorial (110) reflection for analysis because while meridional reflections (00*l*) (such as (002) or (004)) give intensity on a 2D detector only for certain specific sample orientations with respect to the primary beam, equatorial reflections (*hk*0) are always present due to the fibre symmetry of the fibrils. The (110) reflection of the fibre will form a ring with radius q_(110)_ approximately in the q_y_ − q_z_ plane, and this ring is denoted hereafter as R110 (Fig. 2c1). Other chitin fibres which are oriented at varying angles to the q_x_-axis will form rings which collectively all lie on the 3D sphere of radius q_(110)_ (hereafter *QS110*) (Fig. 2c2,c3).

The chitin nanofibres are packed into planar lamellae with a plywood architecture^26^. It is therefore clear that instead of a single fibre orientation in the scattering volume, there are a range of fibre orientations corresponding to the different sub-lamellar orientations in the lamella (Fig. 2b1–b4). For simplicity, consider initially the principal fibre direction as along the q_x_-axis. Denote the normalized (symmetric) fibre orientation distribution as *w*(*γ*; *γ*_*0*_, Δ*γ*_*0*_), where *γ* is the angle (in the plane of the lamella q_x_ − q_z_) of the fibre long axis with respect to the principal fibre direction, and γ_0_ and Δγ_0_ are the centre and width parameter of the distribution. This distribution leads, in reciprocal space, to a range of R110 rings distributed with the same orientation distribution *w*(*γ*; *γ*_*0*_, Δ*γ*_*0*_). We fix *w*(*γ*; *γ*_*0*_, Δ *γ*_*0*_) to a normalized Gaussian distribution:


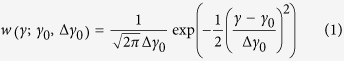


Note that due the lamellar level fibre texture arising from *w*(γ; γ_0_, Δγ_0_), the continuous R110 reflections rings will sum into regions with higher intensity in the pole area of the *QS110* sphere and lower intensity in the equatorial area. The intensities for the rings will constructively add along the vertical q_y_-direction (Fig. 2c2). To write down the intensity distribution on *QS110*, we need an expression for the intensity of the (110) ring on *QS110*, tilted in the q_x_ − q_z_ plane. Considering the (untilted) single chitin fibril oriented along the horizontal q_x_-direction as the reference state, the intensity of the ring will only depend on q_x_, will be peaked at q_x_ = 0, and will decrease rapidly on either side of q_x_ = 0 with a width parameter a_x_. We use the generalized δ-function expression
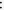



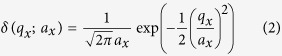


which approaches a sharp band as a_x_ → 0. a_x_ is a parameter characterizing the degree of intrafibrillar alignment of chitin unit cells within a fibril; highly aligned unit cells correspond to small a_x_.

Similarly, the expression for a fibril oriented in the q_x_ − q_z_ plane at an angle γ is


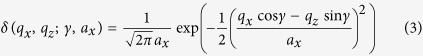


To calculate the intensity at a specific (q_x_, q_y_, q_z_) point on *QS110*, the weighted sum over all individual fibrils leads to


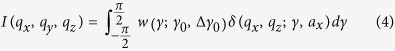


### Incorporation of 3D tilts of fibre lamellar plane

To represent the general case of a plane of fibres tilted out of the (*q*_*x*_ − *q*_*z*_) plane (Fig. 2c3), the tilt can be parameterized by two angles *α* and *β*. The expression Equation (4) holds in the body-fixed frame of the fibril plane (denoted (*q*_*x*_, *q*_*y*_, *q*_*z*_)), where the fibril array is in the *q*_*x*_ − *q*_*z*_ plane. In order to represent the expression in the laboratory-fixed coordinates (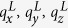; superscript *L* denotes laboratory coordinates), a linear transformation matrix can relate (*q*_*x*_*, q*_*y*_*, q*_*z*_) to (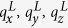). These standard relations are


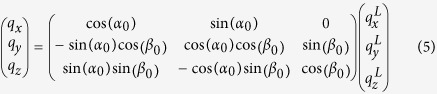


The intensity in laboratory coordinates (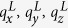) is


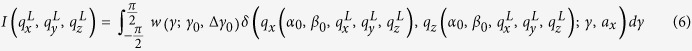


The spherical intensity distribution arising from such a general distribution of fibre orientations is shown in Fig. 2d2, where the regions highlighted on the *QS110* sphere have higher intensity than the rest, owing to constructive addition of intensity from rings. The measured azimuthal intensity profile *I*(*χ*) corresponds to the intersection of the Ewald sphere with *QS110*, as shown schematically in Fig. 2d1.

### 2D intersection of QS110 and Ewald’s sphere

To relate the experimentally measured azimuthal intensity profile *I*(*χ*) to Equation (6) above, we parameterize the wave vectors of the intersection circle located at the intersection of the Ewald’s sphere *Q*_*ES*_ and the *QS110* sphere (Fig. 2d1), the wavevector components on the intersection circle are:


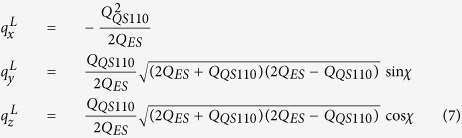


Note the azimuthal angle on the detector is (in usual notation) denoted as χ (Fig. S1, Supplementary Information), Substituting into Equation (6) for (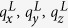), the intensity distribution *I*(*χ*) on the detector is:


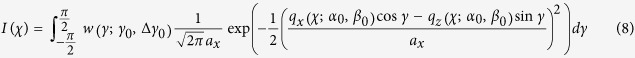


Lastly, our experimental observations show *I*(*χ*) to contain two sets of peaks corresponding to two sets of fibres (IP- and OP-fibres (Fig. 1c5), identified earlier in lobster cuticle^40^,^50^). Both families of fibres (in-plane and out-plane) are characterized by the model in Equation (5), although with different angular parameters and scaling factors (λ_1_ and λ_2_). The experimental data is hence fitted to the sum of two terms of the form of Equation (8).

Equation (8) can be used to fit the experimental *I*(*χ*) curve. In order to find the optimal parameter values (*α*_*0*_, *β*_*0*_, *γ*_*0*_, Δ*γ*_*0*_) and scaling factors (*λ*_*1*_ and *λ*_*2*_), a simple multi-loop over the fit parameters can be carried out numerically, and was our first approach. Computationally, such a simplistic approach is quite slow (with no particular optimizations, the code in Python can take up to one day to fit the parameters with a resolution of 0.1° in *α, β*. Hence, to bring the reconstruction procedure to near real-time performance, we simplified it by taking the limit of Equation (8) to where the width *a*_x_ of the fibre-symmetric diffracting ring for a single fibre goes to zero, enabling an explicit expression for the intensity. This procedure is well justified physically because the limit *a*_x_ → 0 corresponds to a high degree of crystallinity and orientation in an individual fibril, leading to a thin ring (and not a band) of intensity on *QS110*. These individually well-ordered fibrils are assembled into three-dimensional structures as described by the distribution function *w*(*γ*).

### Limiting function for 3D orientation distribution

If we define 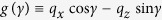, and let a_x _→0, then 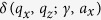 approaches the delta-function, and the following identity for the delta-function can be used:


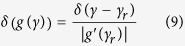


In Equation (9), the prime denotes the derivative and *γ*_r_ is a root of *g*(*γ*). Combining Equation (5) and Equation (7), we find


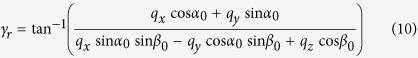


and


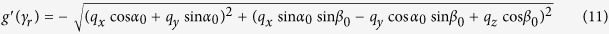


Substituting Equations (9, 10, 11) into Equation (8), the intensity function will become:


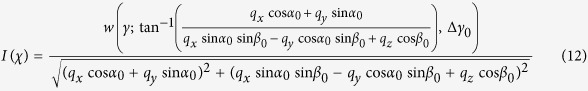


The components *q*_x_, *q*_y_ and *q*_z_ are obtained as functions of *χ* from Equation (7). This function includes no included integral and can hence be used to fit the experimental scattering curves using standard nonlinear fit algorithms such as Levenberg-Marquardt^51^,^52^ with no need for computationally expensive multiparameter minimization methods. In order to account for any residual tilt around the lamellar plane, a numerical convolution of Equation (12) with a Gaussian kernel in β with a narrow width (Δ*β* = 5°) was also found to be useful (as described in *Materials and Methods*).

### Simulated 3D and 2D intensity distributions

To generate simulated diffraction patterns from lamellar distributions with different tilt parameters (*α, β, γ*_*0*_, Δ*γ*_*0*_) in 3D space, we used Equation (6) and (12) to simulate the intensity distribution over the *QS110* sphere and the 2D intensity profile respectively. Such an exercise is useful as it helps provide qualitative indications of how altering (for example) the width of the fibre distribution Δ*γ*_*0*_, or the different tilt angles α and β, may have differing effects on the azimuthal intensity distribution *I*(*χ*).

**Figure 3:**
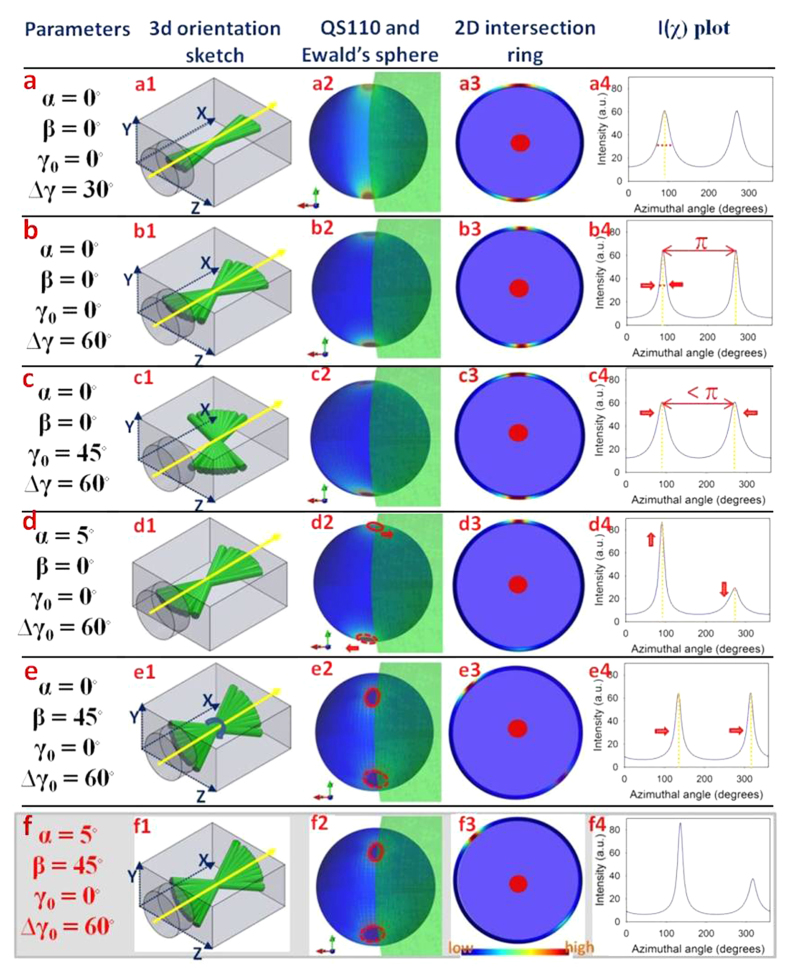
Predicted 3D and 2D diffraction intensity profiles arising from a range of chitin fibre array geometries. (**a1**) Fibre array in q_x_ − q_z_ plane with principal fibre direction along the q_x_ axis. X-ray beam (yellow arrow) along q_x_ direction here and elsewhere in the Figure. (**a2**) 3D scattered intensity distribution on *QS110* arising from the configuration in (**a1**), showing the concentration of intensity at the poles of *QS110* along the q_y_ axis. View is parallel to the q_x_ − q_y_ plane (along the q_z_ axis). Light green shaded region denotes the Ewald’s sphere intersecting *QS110*, and the intersection ring is shown in (**a3**), with the color coding indicating maximal intensities in the vertical direction. (**a4**) Equivalent 2D azimuthal intensity profile *I*(*χ*), showing two equal intensity peaks separated by 180°. (**b1–b4**) Analogous to (**a1**–**a4**), for the case when Δ*γ*_*0*_ increases to 60°, the peak width decreases as explained in the Text. (**c1**–**c4**) Analogous to (**a1**–**a4**), for the case when *γ*_*0*_ is nonzero (45°). As a result, the peak positions between the two peaks of IP fibres become less than 180°. (**d1–d4**) Analogous to (**a1**–**a4**), for the case when *α* is nonzero (5°). As a result, the intensities of the two peaks intersecting the Ewald’s sphere are unequal (upper peak stronger than lower). (**e1–e4**) Analogous to (**a1**–**a4**), but with a nonzero tilt *β* (45°) around the q_x_ axis. In this instance the maxima are rotated around the q_x_ axis, both peaks in *I*(*χ*) are shifted by the same amount and in the same direction (indicated by arrows), but remain equal in height. (**f1–f4**) When both planar tilt angles *α* and *β* are nonzero, the peaks are asymmetric in height, and shifted along the χ-axis.

Figure 3a1–a3 shows the simplest case: an untilted lamella (*α* = *β* = *γ*_*0*_ = 0) aligned along the beam direction. In Fig. 3a2, it is observed that the constructive interference of multiple diffraction rings at different γ lead to a maximum of intensity at the poles of *QS110*, which results in maxima at *χ* = π/2 and 3π/2 in *I*(*χ*) in Fig. 3a3. Interestingly, we observe that when the width of the fibril distribution Δ*γ*_0_ is increased (Fig. 3b1), the width of the two peaks in *I*(*χ*) actually reduces (Fig. 3b3), due to an increased degree of constructive addition of intensity of the diffraction rings at the poles. This would be in contrast to the intuitive expectation that broadening the fibril angular distribution would increase the width of the peaks in *I*(*χ*) and highlights the need for care when considering diffraction from specific crystallographic axes (in this case (110)) in a fibre-geometry. When the principal fibre direction *γ*_*0*_ deviates from along the beam direction (Fig. 3c1), the peak positions in *I*(*χ*) – initially separated by π – come closer together (Fig. 3c3), due to the change in position of the 3D intensity distribution relative to the intersecting Ewald’s sphere seen in Fig. 3c2.

When the lamellar plane tilts in the *xy* plane (*α*-tilt), the amplitudes of the two peaks in *I*(*χ*), originally equal, start to differ (Fig. 3d). The rationale for this change is that the upper pole (along + *y*) of maximum intensity approaches the Ewald’s sphere intersection circle, increasing intensity at *χ* = π/2, while the lower pole (along –*y*) moves further away from the Ewald’s sphere intersection, correspondingly decreasing the intensity at the lower peak at χ = 3π/2. In the complementary direction, a tilt in the *yz* plane (*β*-tilt) leads to a rigid translation of both peaks around the 0 ≤ *χ* ≤ 2π circle (Fig. 3e), with both peaks still separated by exactly π. Lastly, Fig. 3f shows the case where all tilt parameters are non-zero, with all the prior characteristics present (altered width, asymmetric peak heights and peaks separated by less than π).

## Experimental Results and Discussion

**Figure 4:**
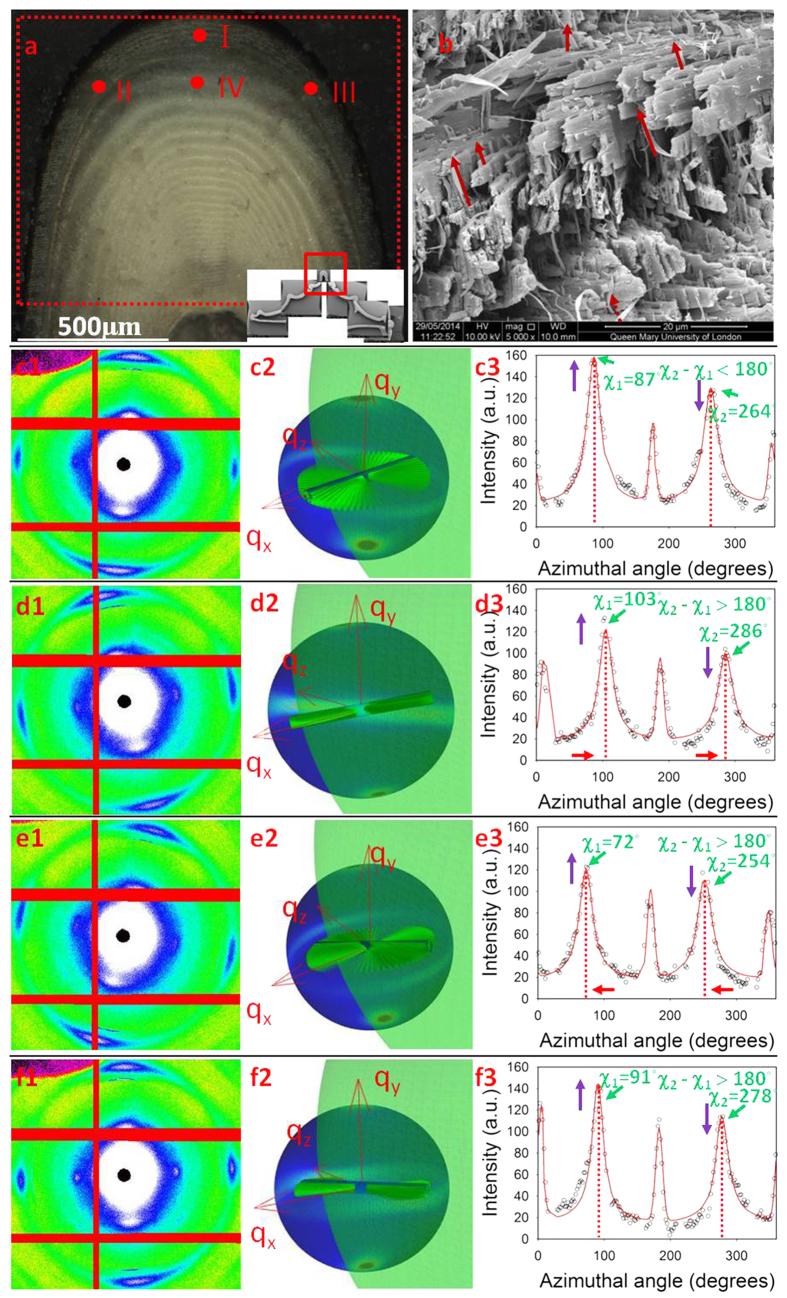
Comparison between experimental X-ray data and model predictions in different locations on the telson cuticle. (**a**) Light microscope image of the adjacent section of the telson sample used in the synchrotron test. The inset image shows a low magnification SEM image of the cross-section of the entire telson. The locations where the example WAXD patterns used for fitting were indicated by I, II, III, IV. (**b**) A SEM image shows the plywood structure formed by the IP fibres and the OP fibres (indicated with red arrows) penetrate from the pore canals. (**c1–f1**) WAXD images collected in synchrotron experiments from location I, II, III, IV respectively, the 110 reflections were located within the read dash rings; (**c2–f2**) Diffraction intensity distributions on *QS110* spheres plotted using the fitted 3D orientation parameters from location I, II, III, IV respectively; (**c3–f3**) Comparison of experimental and fitting *I*(*χ*) curves for each WAXD pattern collected from location I, II, III, IV respectively.

Equation (12) was used to fit the experimental WAXD patterns collected from the central carinae of stomatopod cuticle. From the SEM observations (Fig. 4b, dashed arrows) and prior work on related systems (lobster cuticle)^30^,^40^ we identify two groups of chitin fibres coexisting within the crustacean cuticles. The dominant group is the in-plane fibre (IP) which lies within the plane parallel to the cuticle surface while the other fibre group is aligned along the pore-canals which run perpendicular to the cuticle surface (out-of-plane (OP) fibres). As a result of any variation in orientation (or relative proportion of) the two groups of fibres, the intensity distributions on the *QS110* sphere should change corresponding to the geometrical variation. Fitting representative X-ray diffraction spectra from across the exo- and endocuticle of the telson in the carinal region will provide a first indication of the agreement of the model with experiment, as well as intra-tissue variations.

Four WAXD patterns, collected from different locations (Fig. 4a) of the central carina, are shown in Fig. 4c1–f1. The experimental *I*(*χ*) curves corresponding to the different locations are shown in Fig. 4c3–f3. All the intensity distributions on the *QS110* spheres for different locations are plotted in Fig. 4c2–f2. For clarity, inside the *QS110* spheres the IP fibre planes were plotted using the fitted 3D parameters to give a real-space image of how the fibre orientation distribution affects the intensity distribution on the *QS110* sphere. The corresponding 3D parameters for both IP and OP fibres determined from the fitting procedure for Equation (12) are listed in Table 1. *λ*_*1*_ and *λ*_*2*_ represent prefactors to Equation (12) used in the fit and are proportional to the amount of IP- and OP- fibres present, respectively. The topmost WAXD pattern (Fig. 4c1) was collected on the centreline of the carinae (location I) where the fibre plane is approximately parallel to the *x−z* plane in the coordinate system defined above. The *β* value for the IP fibre is close to 0, indicating that the predicted fibre planes are parallel to the cuticle surface while for the OP fibres the *β* value (86.8°) indicates the fibres are nearly perpendicular to the cuticle surface. As the scanning locations away from the centreline of the carinae, the IP fibre planes are expected to rotate following the curvature of the carinae. As a result, the *β* value will be larger than 0 in the left and smaller than 0 in the right. The *β* value results from location (II) (Fig. 4d1) and (III) (Fig. 4e1) confirms this expectation, with *a, β* value at 17.5° and −15.1° respectively. Also, nonzero α values should be indicative of uneven peaks in the *I*(χ) distributions. This was confirmed by the fitting results in Table 1. As shown in Fig. 4e1, the two peaks of the IP fibres in the location (III) have nearly equal peak intensities; fitting results for the IP fibres in this location give an α value of 0.2°, the smallest among the four locations. As the intensity differences between the two peaks of the IP fibres for the locations (II and IV) get bigger, the *α* values from the fitting results also become larger accordingly. As for the *γ*_*0*_ value, where peak positions (green arrows) were smaller than π (location I), fitted *γ*_*0*_ values are positive, while for the other cases, *γ*_0_ values were negative, resulting in a separation between the two peaks larger than 180°. All *∆γ*_0_ values from I–III were quite large (~75–90°). At these points the beam diameter is comparable to the size of the lamellar layers and hence *∆γ*_0_ characterizes the fibril distribution of the whole Bouligand layer. As the change in angle across a Bouligand layer is 180°, the large *∆γ*_0_ corresponds to a near isotropic distribution of fibrils. In contrast, at IV (centre of carina; endocuticle) the lamella is much larger (3.5 times approximately) than the beam diameter and the beam samples only a part of the Bouligand layer, which has a narrower dispersion than the whole. As a result *∆γ*_0_ is smaller than at I–III. From Table 1 we can further see that the relative amounts of IP and OP fibres (*λ*) also changes at different positions on the telson. Even though the *λ*_1_ and *λ*_2_ values depend on the X-ray transmission at the different positions, the ratio *λ*_2_/(*λ*_1_ + *λ*_2_) is proportional to the OP volume fraction at each position. The distribution of *λ*_2_/(*λ*_1_ + *λ*_2_) values can be used to indicate how the volume fraction of OP fibres changes across the telson sample. It is observed that *λ*_2_/(*λ*_1_ + *λ*_2_) is smallest at point I, which is in the exocuticle, and largest at point IV, which is in the large lamella in the endocuticle at the centre of the carina. These can be linked to differences in pore-canal structure in the two regions: scanning electron microscopy images of fracture surfaces of telson from the exo- and endocuticle show (Fig. S3. Supplementary information) that the exocuticle has a distribution of small pore canals, while in the endocuticle they are larger on average.

**Figure 5:**
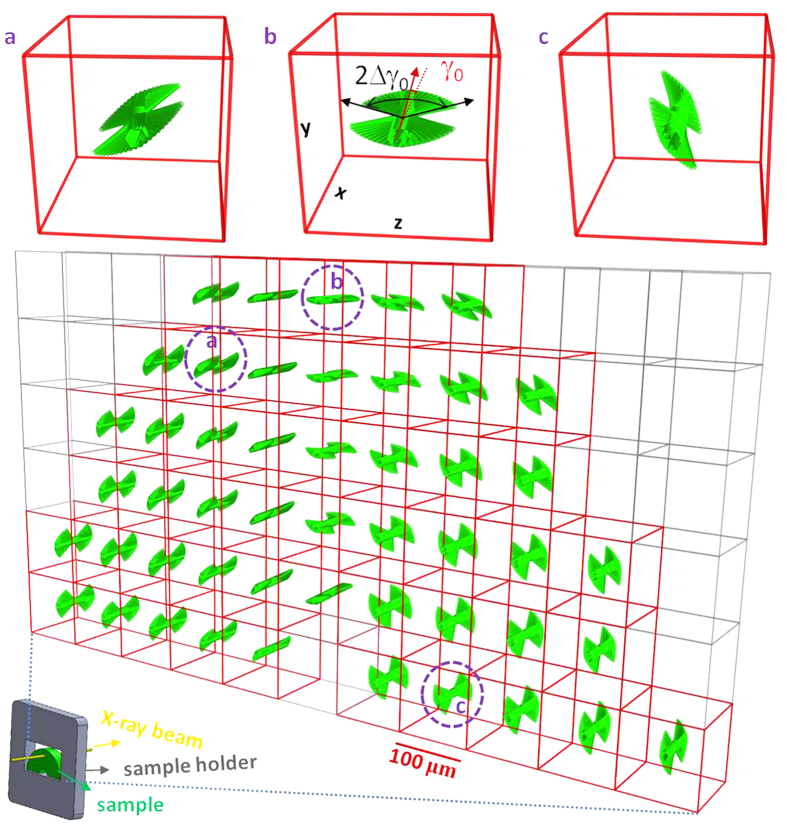
Reconstruction of 3D fibre and lamellar orientation on central carinae of telson from the 2D WAXD scan. 3D fibre orientation distributions across the scanned area (indicated by the red rectangle in Fig. 4a using the fitting results from the 2D WAXD scan. Each point is represented by a lamellar schematic. The mean direction of the lamella is given by the fitted parameters (*α, β, γ*_*0*_) for that point. The width of the lamella is proportional to the fitted value of Δ*γ*_*0*_. Three points were enlarged to show the fibre orientation distributions in 3D more clearly. Only the in-plane fibres are shown for clarity.

The complete 3D map of the chitin fibril orientation distribution across the apex of the telson cuticle is shown in Fig. 5. The apex (denoted as the central carina) was chosen as it is the central reinforcing ridge of the telson^37^. The carina is interesting from a structural-materials point of view as it will be the first site on the cuticle to bear the impact of a predatory or antagonistic force (as, for example, from a competitor stomatopod engaged in intra-species fights^36^). It can be seen in Fig. 5 that the lamellar distribution (representing the Bouligand structure) tracks closely the external shape of the shell. The spatial resolution and interpretation of the technique for reconstructing 3D fibril orientation presented here depends on the relative magnitudes of the X-ray beam diameter, the typical thickness of the Bouligand lamellar thickness, and the sample thickness. In the case considered here, the beam size is of the order of (regions I–III in Fig. 4) or smaller than (IV in Fig. 4) the lamellar thickness. A list of possible size combinations is given in Supplementary Table S1; for most points of the scan shown in Fig. 4, the weight function reported characterizes the average fibril distribution of a Bouligand layer in the zone of the tissue on which the beam is incident.

To study structures with higher spatial resolution than the coarse grid (100 μm × 100 μm with a 10 μm beam) used here, e.g. to reconstruct the fibril distribution within the ~5 μm helicoidal lamellae of the stomatopod saddle spring^53^, a smaller X-ray beam available at synchrotron nanofocus beamlines^54^, combined with sample preparation methods like focused ion beam microscopy to prepare thin sections will be essential. Small-scale (~1–3 μm) variations of fibre angle around the pore canals will broaden the fibre distribution, but the broadening will be negligible when *∆γ*_0_ is much larger than the opening angle around these pores. Further, the existence of other specialized structural motifs such as sensillae^44^ in the scattering volume will need to be accounted for by either including a separate weight function for the fibres in that structure, or by reducing sample- and beam dimensions to interrogate such structures in isolation. We note that this reconstruction is, to the best of our knowledge, one of the first to explicitly quantify the fibril orientation distribution of the Bouligand layered structure^34^, a very common motif in structural connective tissues like bone^5^,^55^ and cuticle^26^,^56^. Specifically, consider a Bouligand layer with thickness *t* and principle fibre direction along the *γ*_0_ direction in-plane, which has been characterized by the technique described here to have a weight function *w*(*γ*; *γ*_0_, Δ*γ*_0_). If the angular resolution between adjacent sublamellae is dγ, the thickness of the sublamella at an angle γ will be:


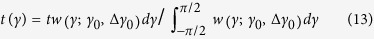


Complementary to high resolution electron microscopic methods^56^ which can provide information in a small (~microns) field of view, scanning microfocus X-ray diffraction combined with 3D reconstruction enables quantitative determination of 3D fibril orientation across millimetre- or larger length scales, within (for the example in Fig. 5) a few (~5) minutes. Further, the method could enable *in situ* tracking of deformation and reorientation of textured biological tissues under external mechanical and environmental stimuli.

## Conclusion

In conclusion, using a novel X-ray diffraction reconstruction model, we have for the first time reconstructed the 3D fibre orientation distribution underlying the chitinous cuticle of a tough, impact resistant biological armour – the stomatopod telson. The reconstruction method takes full account of the complexity in the diffraction pattern arising from the combination of Ewald’s sphere curvature together with a broad fibre distribution in real space. A limiting function approach considerably increased the speed of the fitting process relative to simplistic minimization methods, reducing the time duration from hours to minutes. Avoidance of any requirement for sample rotation in the X-ray beam (and consequent interference between adjacent microvolumes) makes it ideally suited for analysing large areas of scanning microfocus X-ray scattering patterns now being acquired at synchrotron sources. The approach presented here can be generalized to a wide variety of fibre textures in biological and bioinspired composites (for instance by generalizing the fibril distribution function *w*(*γ*)) beyond the planar lamellar structure assumed here.

## Materials and Methods

### Sample preparation

Specimens of peacock mantis shrimp (*Odontodactylus scyllarus*) from the tropical Indo-Pacific were purchased from a commercial supplier (Tropical Marine Centre, London) and stored frozen at −20 °C till use. Before sample sectioning, the telson shells were dissected from defrosted specimens with a scalpel, which were also rinsed in artificial seawater to remove any loose organic debris, and briefly rinsed with deionized water to remove any residual salt. The artificial sea water was prepared using the composition and protocols reported in the literature^57^. For the synchrotron X-ray diffraction measurements, telson shells were sectioned into 1 mm thickness specimens using a low-speed diamond saw (Buehler Isomet, Buehler, Duesseldorf, Germany). Sectioning was done under constant irrigation with deionized water to minimize tissue damage. The samples were sectioned in the plane perpendicular to the long-axis of the specimen, parallel to the medial axis of the telson (Fig. 1). The telsons from three different specimens of comparable size were analysed using the model developed. As the modelling results and fibrillar gradients showed qualitatively similar results for all three telsons, the results for a single telson are presented here.

### X-ray diffraction measurements

Synchrotron wide angle X-ray diffraction measurements were carried out at beamline I22 at Diamond Light Source (Harwell, UK), using 14 keV X-rays with a beam spot focused to ca. 10 μm × 12 μm. Specimens with a 1 mm thickness sliced with a diamond saw were then mounted onto the beamline sample holder in transmission geometry. Transmitted X-ray intensity was recorded using a photodiode detector fixed beyond the sample at the beam stop and normalized by incident intensity measured with an upstream ion chamber. Diffraction data were acquired with a Pilatus P300k-W detector (Silicon hybrid pixel detector, DECTRIS Ltd, Baden-Daettwil, Switzerland). For the composition map shown in Fig. 5, a 7*12 mesh of WAXD patterns were collected to cover the central carina of the nelson with each frame separated by 100 μm in both vertical and horizontal directions. Calibration of the beam centre and sample to detector distance was carried out with silicon powder and the CALIBRANT routine in the data reduction program Fit2D^58^. Azimuthal intensity profiles of *I*(*χ*) for the (110) chitin peak were calculated using the CAKE and INTEGRATE routines in Fit2D (Fig. S2, Supplementary Information). From the experimentally determined 2D WAXD pattern, the azimuthal profile *I*(*χ*) of the (110) reflections is calculated using a wavelength of 0.6888 Å and detector-to-sample distance of 262.3 mm.

### Scanning electron microscopy (SEM)

SEM was used to examine the structural information of the dissected cuticle sample. Fracture surfaces of samples from telson were obtained by gripping samples in a tensile tester and stretching to failure, air dried inside a fume cupboard at room temperature, gold coating, and observing in the SEM (Inspect F, FEI, Eindhoven, Netherlands). Sectioned samples prepared with the diamond saw were also inspected.

### Fitting azimuthal intensity profiles

The experimentally derived *I*(*χ*) was fitted to the model above using customized fitting scripts written in Python (Enthought Canopy, Academic Non-commercial Use License) and using the Matplotlib, Mayavi and *lmfit* Python libraries^52^,^59^,^60^ for both fitting and displaying the data in 3D and 2D. The fitted parameters were (*α*_*01*_*, β*_*01*_*, γ*_*01*_*, ∆γ*_*01*_*, λ*_*1*_) and (*α*_*02*_*, β*_*02*_*, γ*_*02*_*, ∆γ*_*02*_*, λ*_*2*_) for a specific pair of 3D fibre orientations (with the ‘1’ subscript denoting in-plane (IP) fibres and ‘2’ subscript denoting out-of-plane (OP) fibres). The Python functions used for the model functions are mainly from the Scipy stack (www.scipy.org). The routines are in the process of being integrated into the open source synchrotron data evaluation platform Dawn (www.dawnsci.org), with whose development one of us (NJT) is closely involved.

In practice, for fitting Equation (12), the limit of *a*_x_ → 0 will lead to an localized artefactual minimum at along the vertical (*χ* = ±π/2) direction as there is a narrow zone (near the poles of the inverse tangent function for *α, β* = 0) where the regions of high intensity for a very thin ring is closer to the *q*_x_ = 0 origin than the Ewald’s intersection 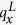. To remedy this, we find a second numerical convolution to account for any residual tilt in the β-direction useful; the physical meaning is that instead of a single principal fibril direction associated with the ring (perpendicular to the diameter and along *γ*_*0*_) there is a fan-shaped distribution of directions in the *yz* plane with a narrow width Δ*β*). In the current work, a numerical discrete convolution of *I*(*χ*) (from Equation (12)) with a Gaussian kernel with Δ*β* = 5° was used to generate the function used in the nonlinear fit; addition of this one-dimensional convolution does not significantly add computational overhead to the fit procedure in contrast to directly minimizing the integral in Equation (8). Another alternative we have explored (with the same effects) is to include higher order terms in 1/*a*_x_ in the expansion of the delta function *δ*(*g*(*γ*)) in terms of a distribution, but this involves a considerable number of additional terms and we do not present it here for concision.

## Additional Information

**How to cite this article**: Zhang, Y. *et al*. Uncovering three-dimensional gradients in fibrillar orientation in an impact-resistant biological armour. *Sci. Rep.* **6**, 26249; doi: 10.1038/srep26249 (2016).

## Supplementary Material

Supplementary Information

## Figures and Tables

**Table 1:** 3D orientation parameters for IP and OP fibres in different positions of the centre carina acquired from asymptotic fitting.

**Location**	**IP fibre (degrees)**					**OP fibre (degrees)**				
	**α**	**β**	**γ**_**0**_	**∆γ**_**0**_	**λ**_**1**_**/(**λ_**1**_** + **λ_**2**_)	**α**	**β**	**γ**_**0**_	**∆γ**_**0**_	**λ**_**2**_**/(**λ_**1**_** + **λ_**2**_)
*I*	0.4	4.7	20.0	83	0.82	2.2	−87.2	88.5	10.0	0.18
*II*	1.0	15.1	−40.0	75.0	0.66	−0.5	−81.0	75.0	10.1	0.34
*III*	0.2	−17.5	−39.1	89.3	0.73	8.5	80.0	−55.0	24.0	0.27
*IV*	1.0	3.4	−47.6	50.0	0.61	−19.0	−86.8	67.3	10.2	0.39
