# Supplementary material for: Uncovering three-dimensional gradients in fibrillar orientation in an impact-resistant biological armour
Source: Sci Rep. 2016 May 23;6:26249. doi: 10.1038/srep26249 (PMC4876318; doi:10.1038/srep26249)
Supplement: Supplementary Information [file srep26249-s1.doc]

**Uncovering three-dimensional gradients in fibrillar orientation in an impact-resistant biological armour**

Y. Zhang^1^, O. Paris^2^, N. J. Terrill^3^ and H. S. Gupta^1,*^

*1. Queen Mary University of London, School of Engineering and Material Science, London, E1 4NS, UK*

*2. Institute of Physics, Montanuniversitaet Leoben, Leoben, Austria*

*3. Diamond Light Source, Harwell Science and Innovation Campus, Harwell, UK*

Corresponding author: Himadri S. Gupta (h.gupta@qmul.ac.uk)

**
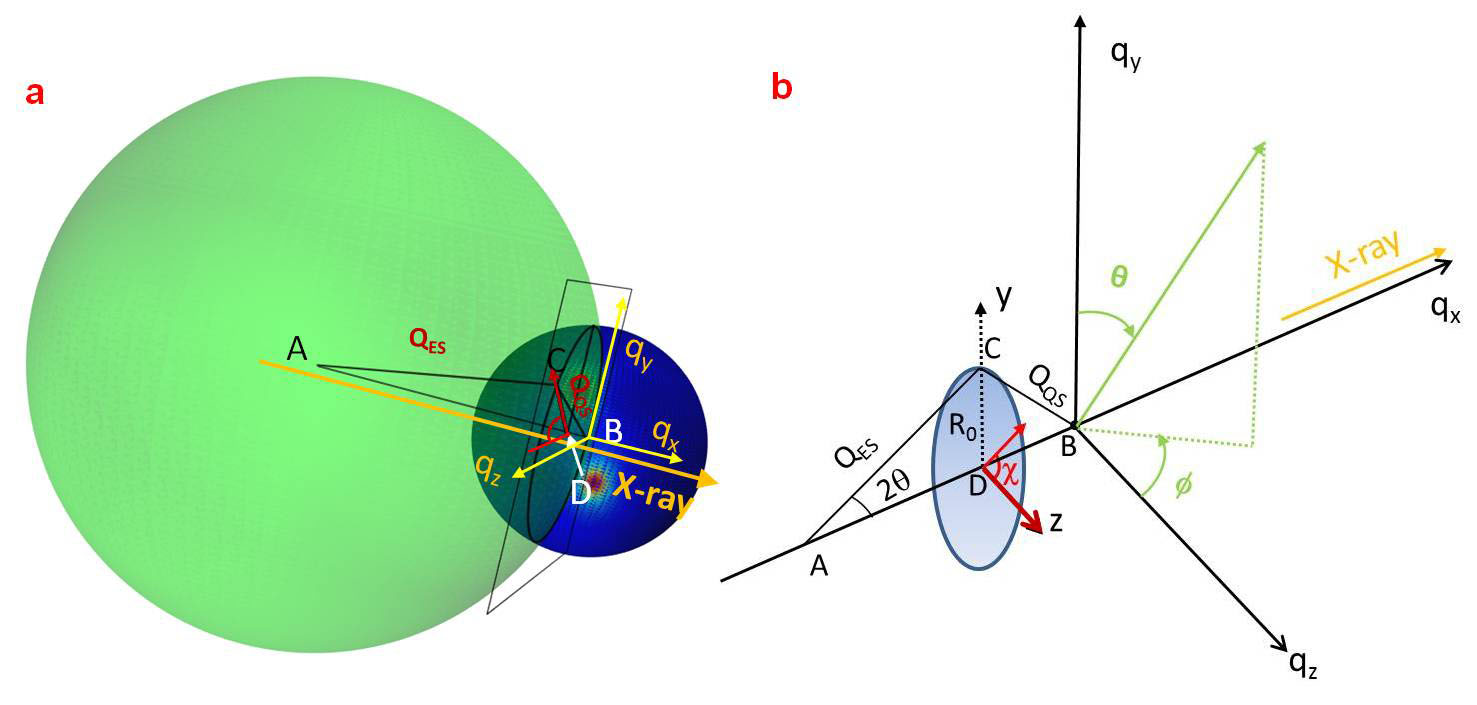
**

**Figure S1. a):** Three dimensional representation, in detail, of the Ewald sphere (light green) intersecting QS110 (dark green), with the intersection circle shown as a dark ring. The rectangle represents the plane (at q_x_ = -λ*q*^2^_(110)_ /(4π)) at which the intersection occurs. The X-ray beam is along the q_x_ direction. The QS110 sphere is centred at the origin A and the Ewald sphere at B. Note that the radius of the QS110 sphere is enlarged 2.5 times for display purpose. **b**): A detail view of A to show the definition of *χ* on the intersection plane of Ewald and QS110 sphere.

**
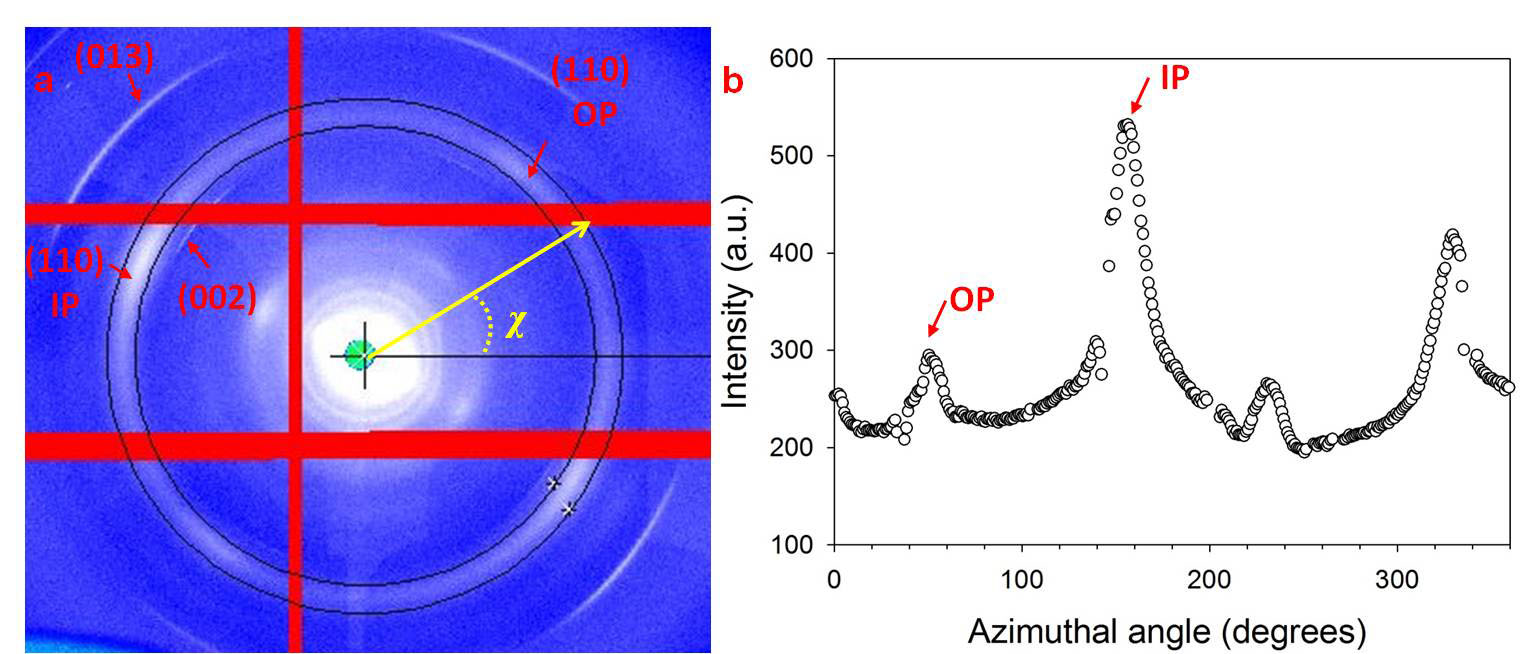
**

**Figure S2**. **a**): Example WAXD pattern collected from stomatopod cuticle with the X-ray beam parallel to cuticle surface. The (002), (110) and (013) reflections were indicated on the pattern. **b**): 1D azimuthal profile of 110 reflections from the pattern in (**a**). The notations “IP” and “OP” represent the components belong to the in-plane fibres and out-of-plane fibres respectively.

**
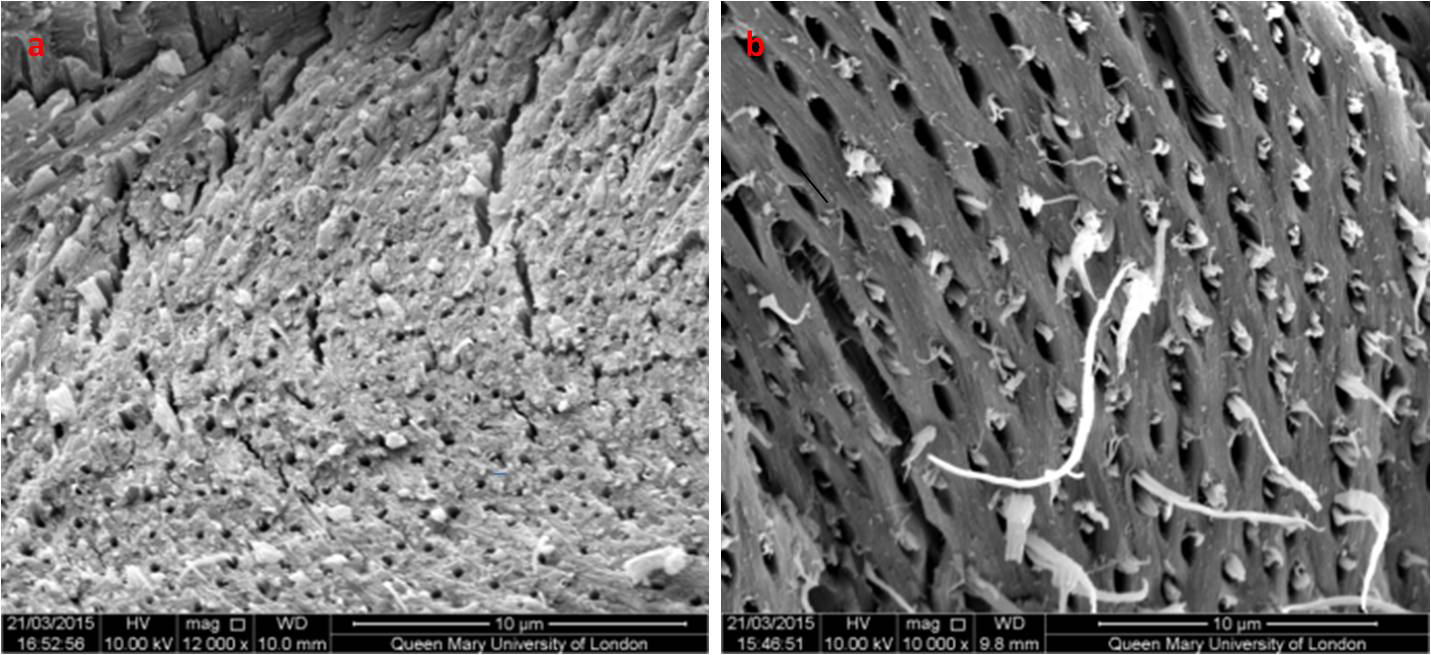
**

**Figure S3**. **a**): SEM image showing the pore canals distribution in the exocuticle. **b**): SEM image showing the pore canals distribution in the endocuticle. In the exocuticle the pore canals are smaller in size (diameter less than 1μm) but more densely distributed, while in the endocuticle region the size of pore canals largely increased to 1-2 μm in diameter but less in number.

**Table S1:** The selection of beam size in relation to the lamellar thickness and sample thickness. The images on the right hand column denote the sample as a set of lamellar layers (Bouligand layers) with an X-ray beam (arrow) passing through in transmission geometry. The height of the sample corresponds to the thickness.

| **Relative dimensions of beam, lamellar width and sample thickness** | **Note** | **Interpretation of w(γ;γ_0_,Δγ_0_)** | **Schematic**  (Note that lamellae do not need to be perfectly ⊥ to the surface; some degree of misalignment will not alter the conclusions) |
| --- | --- | --- | --- |
| Beam size ~ lamellar thickness < sample thickness | Experimental case in this paper (most of points in scan; point I, II and III in Fig. 4) | Averaged fibril distribution of a Bouligand layer in the zone of the tissue | 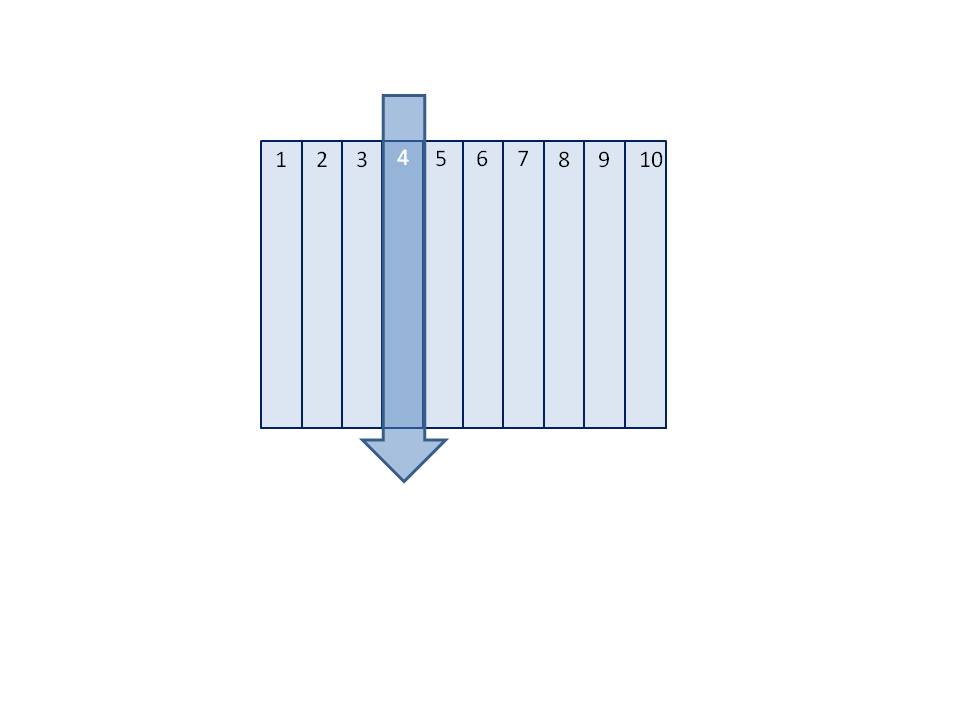 |
| Beam size < lamellar thickness < sample thickness | Experimental case when considering the wide lamellae in the central carinal endocuticle near the boundary to the exocuticle (point IV in Fig. 4) | Fibril distribution in a sublamella of the Bouligand layer, (unless the angle of the lamella is very far from ⊥ to the surface plane) | 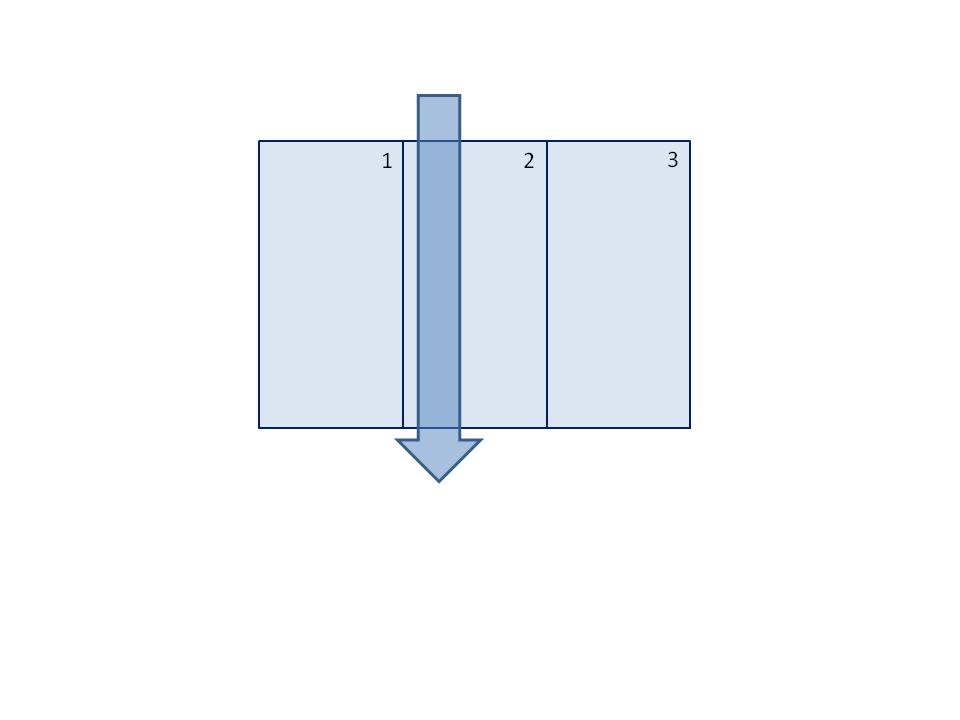 |
| Beam size < lamellar thickness ~ sample thickness | Using a nanofocus X-ray or microfocus beam with diameter ~ 1 micron | Fibril distribution in a sublamella | 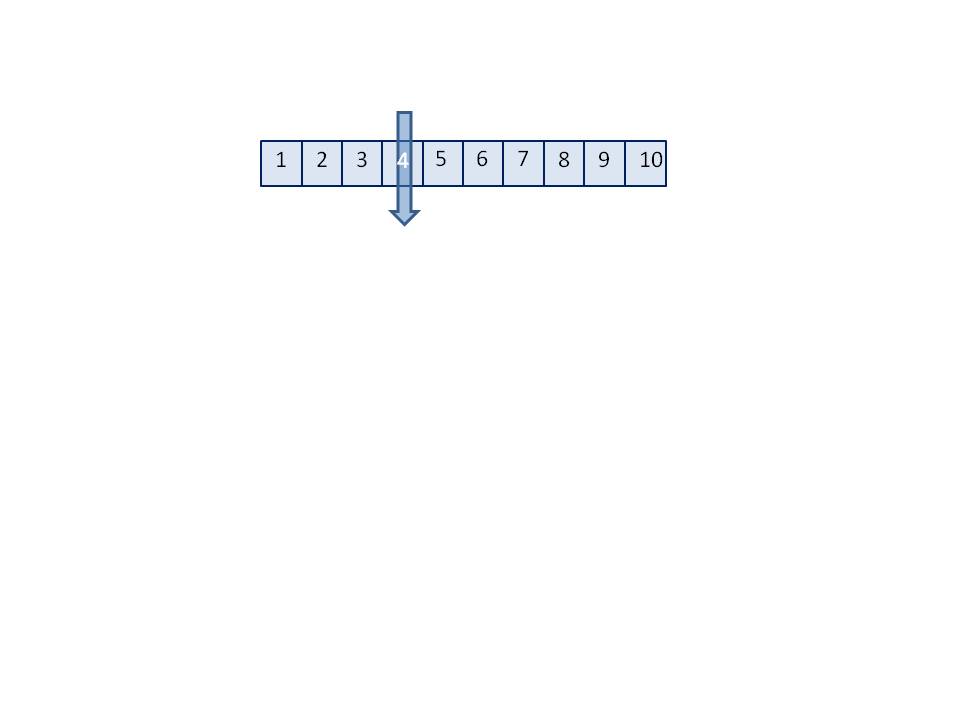 |
| lamellar thickness < beam size ~ or < sample thickness |  | Averaged fibril distribution of a Bouligand layer in the zone of the tissue | 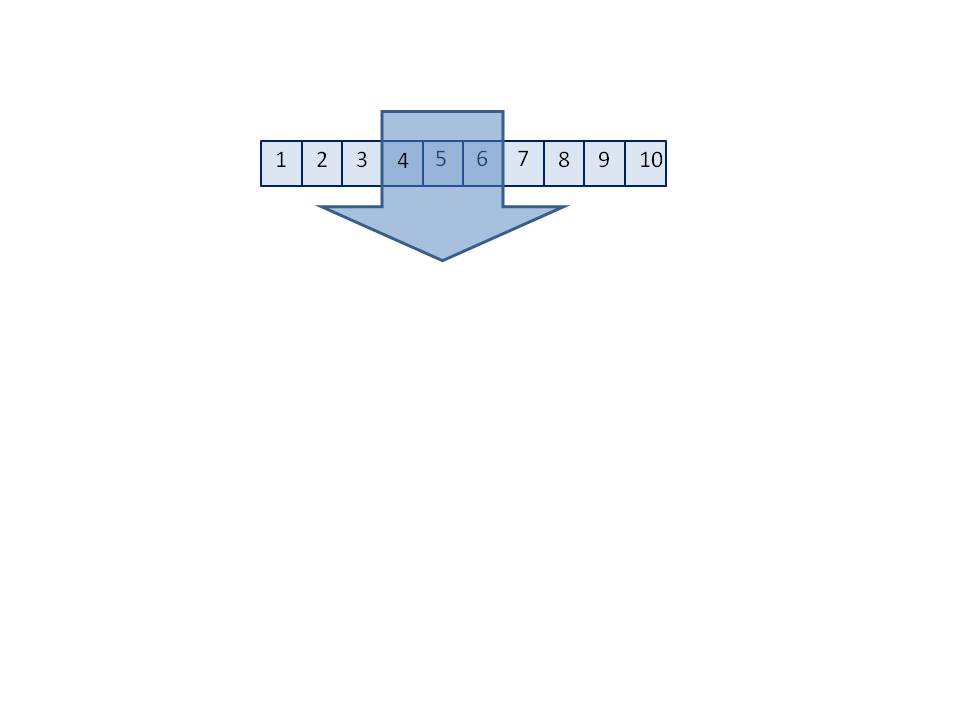 |
